# Retinal Displacement Following Vitrectomy for Rhegmatogenous Retinal Detachment: A Systematic Review of Surgical Techniques, Tamponade Agents, and Outcomes

**DOI:** 10.3390/jcm14010250

**Published:** 2025-01-03

**Authors:** Paulina Siwik, Tomasz Chudoba, Sławomir Cisiecki

**Affiliations:** 1 National Medical Institute of the Ministry of the Interior and Administration, 02-507 Warsaw, Poland; tomasz.chudoba@cskmswia.gov.pl; 2 Department Ophthalmology, Miejskie Centrum Medyczne Jonscher, 93-113 Łódź, Poland; cisieckislawomir@gmail.com

**Keywords:** rhegmatogenous retinal detachment, pars plana vitrectomy, retinal displacement, tamponade agents, postoperative positioning, visual distortion

## Abstract

**Background:** Rhegmatogenous retinal detachment (RRD) is a severe condition that may lead to permanent vision loss if untreated. Pars plana vitrectomy (PPV) has become a preferred surgical intervention, particularly in complex cases. Objective: Retinal displacement (RD) following PPV for RRD can lead to visual distortions and can negatively impact patient quality of life. This review examines surgical techniques, tamponade choices, and postoperative strategies to mitigate displacement risks and their clinical implications. **Methods:** A systemic review of studies from 2010 to 2024 was conducted using PubMed, MEDLINE, and Ovid. The search included terms such as “retinal displacement, “tamponade agents”, and postoperative positioning”. Inclusion criteria focused on studies addressing PPV outcomes, retinal alignment, and visual distortions. Methodological quality was assessed using PRISMA guidelines. **Results:** Gas tamponades were associated with higher RD rates compared to silicone oil. Intraoperative use of perfluorocarbon liquid (PFCL) improved retinal stability. Postoperative positioning strategies significantly reduced visual distortions. **Conclusions:** Surgical and postoperative techniques substantially influence RD risk. Advances in imaging and tamponade agents offer promising avenues to improve patient outcomes and minimize RD.

## 1. Introduction

**Figure 1:**
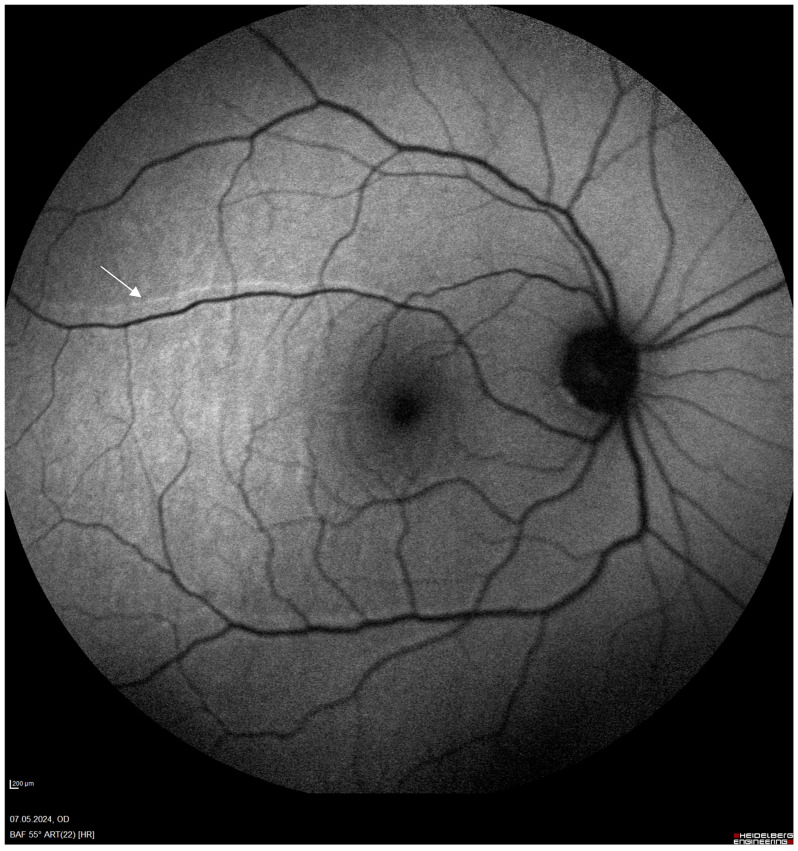
Retinal vessel printings on fundus autofluorescence examination (white arrow).

Rhegmatogenous retinal detachment (RRD) is a separation of neurosensory retina from the retinal pigment epithelium due to a full-thickness retinal break that allows fluid from the vitreous cavity to accumulate beneath the retina, resulting in detachment [1]. If untreated, this condition can lead to permanent vision loss, highlighting the importance of prompt surgical intervention. RRD has an estimated incidence of 6.9–18.2 cases per 100,000 people annually, with a higher prevalence among individuals with risk factors such as high myopia, lattice degeneration, ocular trauma, and both uncomplicated and complicated phacoemulsification [2]. Pars plana vitrectomy (PPV) has become the preferred surgical approach over scleral buckling and pneumatic retinopexy. Although meta-analyses of prospective, randomized, and controlled trials comparing PPV and scleral buckling reveal no significant difference in primary reattachment rates, many specialists currently favor PPV for managing uncomplicated RRD, particularly in complex cases where other methods may be less effective [3]. The main purpose of PPV is to relieve vitreous traction, remove residual vitreous, and seal retinal breaks, thereby achieving reattachment [4]. One of the significant complications following RRD repair is unintentional retinal displacement, where the retina reattaches but with a positional shift, potentially leading to visual distortions, such as metamorphopsia. This phenomenon, marked by the misalignment of photoreceptor cells, can be visualized using blue-fundus autofluorescence imaging (B-FAF). Dell’Omo et al. introduced the term “retinal vessel printings” [Figure 1] for this phenomenon, while Lee at al. refereed to it as “retinal ghosts” [5,6]. The choice of tamponade agents and intraoperative stabilization techniques has been evaluated in a limited number of studies as a potential factor in retinal displacement risk [6,7,8]. Gas tamponades, such as sulfur hexafluoride (SF6), perfluoropropane (C3F8), and perfluoroethane (C2F6), are characterized by their short absorption times, which facilitate relatively quick visual recovery and make them effective in primary, uncomplicated RRD cases, including those involving the inferior quadrants [9]. On the other hand, silicone oil (SO) is commonly used in complex cases, such as those involving proliferative vitreoretinopathy (PVR), recurrent detachment, and retinal detachment associated with giant tears, diabetes, or trauma. However, SO presents potential disadvantages, including a re-detachment rate of up to 16% after removal, along with a risk of cataracts, increased intraocular pressure, keratopathy, cystoid macular edema, epiretinal membrane formation, and especially emulsification, which is more common in light-chain SO [10]. Perfluorocarbon liquid (PFCL) served as a temporary intraoperative tamponade that facilitates retinal stabilization during PFCL-assisted drainage. It is typically replaced with SO at the end of the surgery. In cases involving high-rate PVR, a double filling technique with PFCL and silicone oil (SO) is sometimes employed as a short-term postoperative tamponade, remaining in the vitreous cavity for five days to two months to obtain adequate chorioretinal laser scars, which reportedly develop within two weeks post surgery. This technique is especially valuable in cases of inferior retinal detachment, as it helps prevent postoperative PVR, though the potential for chemical and mechanical toxicity and the risk of PFCL retention must be considered [4,11]. Advances in imaging techniques, such as b-FAF and optical coherence tomography (OCT), have enabled clinicians’ ability to assess retinal positioning with precision. These methods can detect subtle hyperfluorescent vessels that run parallel to retinal blood vessels, indicating increased metabolic activity in areas of retinal pigment epithelium that are acutely exposed to light due to the displacement of retinal vessels from their original locations. Some studies suggest that wide-field imaging may be a more valuable tool for detecting minor displacements that traditional imaging might overlook, thereby offering a more comprehensive assessment of retinal alignment [1,8]. This systematic review seeks to consolidate and evaluate current evidence on the role of preoperative assessment, tamponade choices, and postoperative management strategies that influence retinal displacement in patients undergoing PPV for RRD. By synthesizing these findings, we aim to identify effective approaches for minimizing displacement and enhancing patient outcomes, ultimately contributing to the foundation of optimized surgical protocols for managing RRD.

## 2. Methods

**Figure 2:**
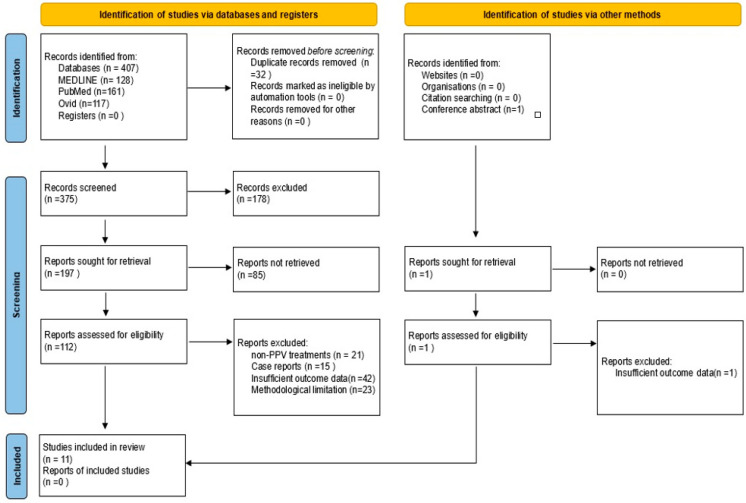
PRISMA 2020 flow diagram.

This review included studies examining postoperative retinal displacement as an outcome following PPV for RRD. Eligibility criteria included patients undergoing PPV for RRD, without restrictions on age or demographics. Studies were required to focus on PPV surgical techniques, including comparisons of tamponade agents such as SF6, C3F8, silicone oil, and PFCL. Quantitative measures of retinal displacement, visual distortions, and postoperative best-corrected visual acuity (BCVA) were considered. The review included only randomized controlled trials, observational studies, and cohort studies, while excluding studies focused on non-PPV treatments, case reports, and case series with insufficient outcomes or methodology data. A comprehensive search was conducted in the PubMed, MEDLINE, and Ovid databases alongside manual searches of gray literature repositories such as conference abstracts. The search strategy, including keywords and Boolean logic, was applied with filters for English language, publication year (2010–2024), and clinical trials. Keywords included “retinal displacement”, “rhegmatogenous retinal detachment”, “pars plana vitrectomy”, “PPV”, “tamponade agents”, “perfluorocarbon liquid”, “visual distortion”, “metamorphopsia”, and “postoperative positioning”. The review was not registered in any systematic review database or registry. However, compliance with Preferred Reporting Items for Systematic Reviews and Meta-Analyses (PRISMA) guidelines was maintained throughout the review process to ensure transparency and standardized reporting, despite the lack of formal registration. Titles and abstracts of all retrieved studies were independently screened by two reviewers to assess eligibility. In cases of ambiguity, full-text articles were obtained for further review. Discrepancies in study selection were resolved by a third reviewer. The PRISMA flow diagram (Figure 2) illustrates this process, showing records identified from MEDLINE, PubMed, and Ovid databases totaling 407, with 128 from MEDLINE, 161 from PubMed, and 117 from Ovid. After removing 32 duplicates, 375 unique records were screened. Then, 178 records were excluded based on title and abstract screening, leading to 197 reports sought for full-text retrieval. Of these, 85 reports could not be retrieved. One conference abstract from the 21st European Vitreo Retinal Society Meeting was assessed for eligibility and excluded due to insufficient data.

A total of 112 full-text reports were assessed for eligibility, resulting in 11 studies that met the criteria for inclusion, while 101 reports were excluded due to non-PPV treatments (*n* = 21), case reports (*n* = 15), insufficient outcome data (*n* = 42), and methodological limitation (*n* = 23). Data items extracted from each study included study type, publication year, geographic location, patient demographics, number of eyes treated, tamponade agents used, PFCL use, and follow-up duration. Primary outcomes focused on retinal displacement, while secondary outcomes included postoperative BCVA, visual distortion, and direction of retinal displacement. Imaging techniques for measuring displacement, such as fundus autofluorescence (FAF) and optical coherence tomography (OCT), were also recorded. The methodological quality of each study was independently assessed by two reviewers using the Risk Of Bias in Non-randomized Studies-of Interventions (ROBINS-I) [Table 1] framework for observational studies and the Cochrane Risk of Bias tool [Table 2] for randomized controlled trials [12,13]. Studies with a high risk of bias were excluded from the final synthesis. Any conflicts of interest and industry sponsorship were documented for transparency. Only direct evidence was considered in the analysis, and the overall quality of evidence was evaluated using the Grading of Recommendations, Assessment, Development, and Evaluations (GRADE) framework [Table 3]. A narrative synthesis was conducted as statistical meta-analysis was not feasible due to heterogeneity across the included studies. Potential limitations of this review include its lack of registration, which may affect reproducibility, and the exclusion of non-English-language studies. Additionally, reliance on published data may introduce publication bias. This comprehensive approach, with adherence to PRISMA guidelines and the inclusion of a detailed search strategy, flow diagram, and risk of bias assessments, ensures a transparent and rigorous assessment of the available evidence on postoperative retinal displacement following PPV for RRD.

## 3. Results

A total of 407 studies were initially identified. After removing duplicates, the number of studies was reduced to 375. Of these, 252 were excluded for not meeting eligibility criteria. An additional 112 studies were excluded following a full-text review, primarily due to their focus on alternative treatments rather than on PPV for RRD. Ultimately, 11 studies met the inclusion criteria and were included in the final review. The selection process is illustrated in a PRISMA flow diagram (Figure 2). The included studies varied in design, including randomized controlled trials (*n* = 2), observational studies (*n* = 7), a retrospective study (*n* = 1) and interventional case series (*n* = 1). Sample sizes ranged from 20 to 239 participants, with patient demographics generally reflecting the broader population affected by RRD. Table 4 summarizes the characteristics of each study, including study type, number of participants, intervention types, age, gender, number of centers, year, follow-up duration, PVR grade, macula status, extent of detachment, intraoperative PFCL use, instances of retinal displacement, postoperative positioning, and tamponade agent use. The comparison is illustrated in Table 4.

The primary outcomes are as follows. 

### 3.1. Presence of Retinal Displacement

**Figure 3:**
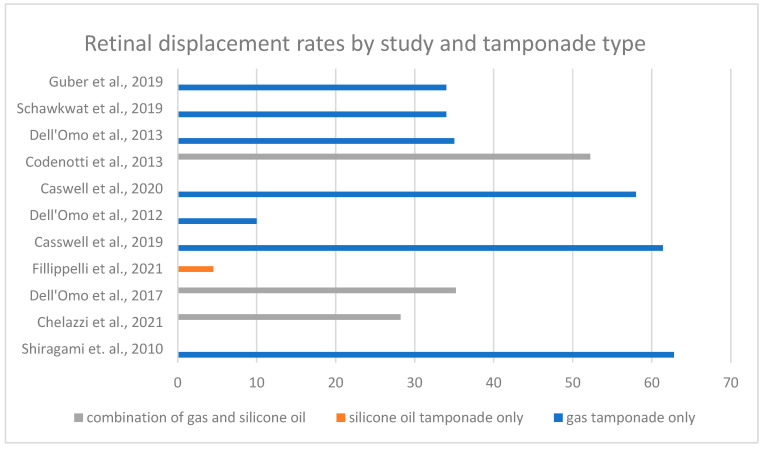
The diagram of primary outcomes with the characteristic of tamponade used [3,4,5,7,8,14,15,16,17,18,19].

The primary outcome was the presence of retinal displacement following PPV for RRD. Retinal displacement, defined as the postoperative misalignment of retinal photoreceptor cells, detectable on fundus autofluorescence, varies in included studies from 4.5% to 62.8% (Figure 3). The results were influenced by inclusion criteria, surgical techniques, tamponade selection, and postoperative management strategies.

### 3.2. Impact of Tamponade Agents

The type of tamponade used was examined in four studies. Gas tamponade (SF6, C3F8) was used in ten studies [3,4,5,7,8,14,16,17,18,19], while SO was used in four. Three of these studies allowed for either gas or SO [4,7,8], and one study exclusively used SO [15]. Filipelli et al. investigated patients who underwent PPV with SO injection, and found that only 2 (4.5%) of 44 eyes experienced retinal displacement, suggesting that SO injection might provide significant stabilization [15]. Dell’Omo et al. reported a notable difference in retinal displacement rates, with 41.2% of eyes with gas tamponade experiencing displacement compared to only 14.3% of eyes with SO (*p* = 0.009). In this study, the type of tamponade was identified as the only significant predictor of retinal displacement (odds ratio, 5.3; *p* = 0.007) [8]. Similarly, Codenotti et al. reported a displacement rate of 71.4% in eyes with gas tamponade compared to 22.2% in eyes with SO tamponade [7]. In another study type, gas tamponade did not affect retinal displacement rates (OR, 0.70; 95% CI; *p* = 0.73); however, no other tamponade agents were included in this analysis [16]. Additionally, one study concluded that the specific type of gas used did not significantly impact retinal displacement [19].

### 3.3. Preoperative Macula Status

Macula-off status was an inclusion criterion in seven studies [3,8,15,16,17,18,19], while four studies enrolled patients regardless of macula status [4,5,7,14]. Additionally, one study concluded that macula-off RRD was associated with a higher risk of retinal displacement, showing a statistically significant impact (*p* = 0.016) [14]. Chelazzi et al. [4] reported that among 71 patients, 28.2% (11 of 39) experienced retinal slippage; notably, all patients had macula detachment, and none of the 32 macula-on patients experienced retinal displacement. This finding was consistent with other studies.

### 3.4. Extent of Detachment in Quadrants

Seven studies reported the number of quadrants involved in retinal detachment [5,7,8,14,15,18,19]. Only one study found a significant impact of quadrant involvement on retinal displacement (*p* = 0.019) [14]. In four studies, the extent of retinal detachment did not significantly affect the rate of displacement [8,15,18,19]. However, it should be noted that these studies included a limited number of involved retinal detachments quadrants and were not designed to assess this factor as a primary outcome.

### 3.5. Proliferative Vitreoretinopathy

Four studies specified PVR grading, but only two included patients with grade C PVR [4,8,15,16]. Three out of eleven studies excluded cases with any PVR [14,18,19]. In one study involving 44 eyes, retinal vessel printing were observed in only 2 eyes (4.5%). Both had PVR B and in both cases intraoperative PFCL was used with fluid–air exchange prior to SO injection [15]. The role of PVR was not reported across the remaining studies.

### 3.6. Effectiveness of Perfluorocarbon Liquid

The use of PFCL as an intraoperative stabilizing agent was evaluated in four studies, while eight studies reported its use overall [5,7,8,14,15,16,18,19]. In one study, PFCL was suggested as a temporary stabilizing factor that enhances retinal stability during surgery, though this finding was not supported by specific statistical data [15]. Two studies observed significantly smaller postoperative macular shift with PFCL use (*p* = 0.049) [18,19]. Conversely, one study hypothesized that PFCL may not be necessary for stabilization [4]. In a study by Dell’Omo et al., PFCL was used in 80% of cases, but no significant effect on retinal displacement was observed [8].

### 3.7. Age Effect

Two studies found that patients younger than 60 years were at a greater risk for postoperative retinal slippage, with a statistically significant association (*p* = 0.03) [18,19].

### 3.8. Effect of Detachment Duration

One of the studies examined the impact of detachment duration on retinal displacement, suggesting that detachment duration does not significantly affect displacement (OR 1.02; 95% CI; *p* = 0.74) [16]. However, this finding was based on patients with a history of 2–6 days of detachment duration [16]. Dell’Omo et al. reported similar outcomes in a group with detachment duration of 8–9 days [8]. In most studies, preoperative detachment duration ranged from 1 to 9 days [16,17]. Only one study included patients with a longer history of symptoms. Consequently, there are insufficient data to analyze the impact of detachment duration on retinal displacement comprehensively.

### 3.9. Postoperative Management and Head Positioning

Postoperative positioning protocols were included in ten studies [3,4,7,8,14,15,16,17,18,19]. Several reports emphasized the importance of head positioning following surgery. Dell’Omo et al. concluded that strict, 2 h face-down positioning immediately after PPV with gas tamponade could reduce the incidence of retinal displacement to 35% in macula-off patients, a lower rate than previously reported [3]. Casswell et al. compared displacement outcomes at 6 months and 8 weeks, noting an increased risk of retinal displacement in patients positioned to support the break compared to those in the face-down group within the first 24 h at the 6-month follow-up (OR 1.77; 95% CI; *p* = 0.04). In the face-down position, an average displacement of 0.5 degrees was observed, compared to 0.8 degrees in the support-the-break position within the first 24 h, as measured at the 8-week follow-up [16]. Another study compared the log-roll position to a flat-lying position for six hours, followed by positioning according to the location of the break for seven days. Retinal displacement rates were 46.2% in the log-roll group and 20.8% in the flat-lying group, with no significant difference in macular shift associated with postoperative positioning (*p* = 0.94). Both groups exhibited similar degrees of retinal shift when rotation occurred [18,19]. Chelazzi et al. concluded that postoperative supine positioning in cases of macula-off RRD with residual submacular fluid at the end of the operation reduces the risk of retinal displacement [4].

### 3.10. Image Modality

In the literature, only one study compared the influence of imaging modalities on retinal displacement findings using fundus FAF [17]. Casswell et al. examined 70 patients using both fundus camera (FC) and confocal scanning laser ophthalmoscopy (cSLO) with 50- and 55-degree images. Vessel shift was detected at a slightly higher rate with FC (61.4%) compared to cSLO (52.8%), though they reported no significant difference between the two techniques [17]. Overall, most studies presented in this review have shown a preference for using the cSLO technique.

### 3.11. Postoperative Subretinal Fluid

Chelazzi et al. asserted that anatomical retinal reattachment can be achieved without the complete drainage of subretinal fluid, as residual fluid can be absorbed by the retinal pigment epithelium during the initial postoperative hours. It is believed that complete drainage of subretinal fluid is essential in patients undergoing SO endotamponade to prevent PVR formation and the recurrence of RRD [4]. Dell’Omo et al. suggested that small amounts of residual subretinal fluid at the end of surgery do not play a critical role in preventing displacement [8]. However, patients with residual subretinal fluid in the macular region were excluded from this study [8].

The secondary outcomes are as follows.

### 3.12. Distortion

Four studies reported significant visual distortion associated with retinal displacement, with all affected patients presenting with macula-off status [4,16,18,19]. Chelazzi et al. noted distorted vision in 8 out of 11 patients with retinal slippage [4], while Schawkat et al. reported metamorphopsia in 10 out of 17 patients with retinal shift [18].

### 3.13. Postoperative Best Corrected Visual Acuity

Four studies found that retinal displacement did not affect the postoperative logMAR best corrected visual acuity (BCVA) [4,8,18,19]. Caswell et al. reported worse BCVA in patients with retinal displacement; however, it was noted that the study might not be sufficiently powered to detect a true clinical difference [16].

### 3.14. Direction of Displacement

The direction of retinal displacement was reported in four studies [7,8,14,16]. Dell’Omo at al. observed downward displacement in 39 cases (88.6%) and upward in 5 cases (11.4%). This was independent of the location of detachment. Downward displacement occurred with higher incidence in cases of inferior detachments (41.7%) compared to superior detachments (22.6%). Additionally, upward dislocation occurred only in cases of superior or combined superior and inferior detachments. However, no significant correlation was found between the direction of retinal displacement and the localization of retinal detachments (*p* = 0.09) or retinal breaks (*p* = 0.29), nor was there any dependency on the type of the tamponade used [7,8]. Shiragami et al. also reported downward retinal displacement in 62.8% of patients [14]. Casswell et al. linked the amplitude of displacement with higher distortion on the D chart (*p* = 0.008) [16].

## 4. Discussion

Retinal displacement was first reported in 2010 by Shiragami et al. in 62.8% of patients. This finding was connected to increased metabolic activity in the retinal pigment epithelium, previously shielded by the retinal vessels and then acutely exposed to light postoperatively due to vessel displacement from their original positions [3]. Long-term effects of retinal displacement remain underexplored. Displacement has been linked to persistent visual disturbances, such as metamorphopsia, which can significantly impair patients’ ability to perform daily tasks. Furthermore, the psychological burden associated with these distortions often impacts the quality of life, emphasizing the importance of addressing this issue in future research. The main aim of this review was to identify factors that contribute to retinal displacement and to examine their influence on postoperative outcomes. Our findings suggest that the choice of tamponade agent is a crucial determinant of postoperative retinal positioning. One hypothesis is that SO injection helps move the residual subretinal fluid (SRF) toward retinal breaks during air–fluid exchange, thereby reducing the likelihood of retinal slippage. Another theory attributes this effect to the different physical properties of SO and gas tamponades [3]. However, these results should be interpreted cautiously due to limited data and the lack of randomized comparative studies. Additionally, RRD cases treated with SO injections tend to be more complex, potentially impacting outcomes. A lower incidence of detectable displacement may also be related to longer intervals from the onset of detachment, potentially resulting in changes in the retinal pigment epithelium (RPE) and subsequently reducing postoperative hyperfluorescence on FAF images [15]. In most studies, macula-on status was used as an exclusion criterion due to the clinical insignificance of peripheral retinal shifting on visual acuity. Furthermore, anatomical reattachment of the retina does not always lead to complete visual recovery. Metamorphopsia is one of the most commonly reported postoperative complaints. Dell’Omo et al. demonstrated that the presence of outer retinal folds was an independent predictor of metamorphopsia. Additional predictors of postoperative visual distortion include younger age, preoperative macula-off status, disruption of the external limiting membrane, and the presence of SRF [20]. Given above, these factors should be considered in studies measuring distortion. Another limitation is that some studies did not use meticulous or reliable tools to measure visual distortion, highlighting the need for more precise and standardized assessment methods. Zhou et al. observed that younger patients are more prone to long-standing RRD with persistent and dense SRF, which is challenging to drain and may lead to persistent SRF. This may explain why younger age is associated with a higher incidence of retinal displacement. PVR is another variable worth exploring, as it involves cellular membrane growth and contraction within the vitreous cavity and on the retinal surface, leading to fibrosis. Currently, the literature lacks sufficient data to determine whether PVR grade is associated with retinal displacement. Given its potential impact on retinal flattening, further research into this topic is warranted [21]. Additionally, PFCL used as a temporary intraoperative tamponade is thought to stabilize the retina effectively during surgery, especially in macula-off detachments where precise alignment is critical. PFCL helps displace fluid anteriorly, enabling more complete subretinal drainage and potentially reducing the need for posterior drainage retinotomy [22]. PFCL may improve postoperative retinal positioning, potentially enhancing visual outcomes. However, PFCL’s benefits may vary based on patient-specific factors and surgical conditions, underscoring the need for individualized surgical planning. Conversely, PFCL carries risks such as retinal toxicity, RPE atrophy, reduced retinal sensitivity, inflammation, membrane formation, recurrent RD, and corneal endothelial damage [4]. An intriguing area of research not yet extensively explored is the impact of heavy oil tamponades on retinal displacement. Some studies suggest that heavy oil may eliminate the need for strict postoperative positioning, which could be beneficial in select cases [23]. Notably, the extent of detachment does not appear to influence retinal displacement [18]. This finding, however, should be interpreted with caution due to small sample sizes, and the limited representation of patients with varying numbers of detached quadrants in each study [18]. Similarly, the location of detachment or break did not appear to impact retinal slippage [8,14,16]. Only one study examined the impact of the modality of exchange, but the findings should be cautiously interpreted due to the small sample size and lack of a randomized trial [15]. The literature does not support the notion that complete removal of SRF improves postoperative anatomical outcomes; however, it remains a significant factor in retinal shift and subsequent visual outcomes [4]. Retinal slippage is associated with small amounts of residual SRF that persist immediately post-operation, allowing the retina to shift inferiorly under gravity if the patient adopts an upright position [22]. Cobos et al. identified residual SRF as the most significant cause of retinal displacement [24]. Furthermore, persistent SRF in the macula for up to 6 months is significantly associated with poor functional recovery after macula-off RRD surgery, likely due to impaired metabolic function of the photoreceptor–RPE complex [20]. Another issue is the variability in FAF imaging techniques and retinal field sizes across studies [17]. Casswell et al. hypothesized that the FC uses a longer excitation wavelength (530–580 nm) compared to cSLO imaging (488 nm), which may affect retinal displacement detection by exciting fundal fluorophores differently. Despite this, outcomes with both modalities are comparable, though with only moderate agreement [17]. Posterior retinotomy is another factor potentially influencing visual outcomes, as it may be associated with PVR and visual defects [4]. One study found that drainage through posterior retinotomy was not associated with retinal displacement; however, this factor needs further investigation [16]. Postoperative head positioning is a major factor influencing retinal slippage. All studies included postoperative positioning protocols, emphasizing its importance. However, data remain inconclusive regarding the most beneficial positioning approach, although the upright position is generally presumed to be unfavorable. While most studies indicate that retinal displacement does not significantly impact visual acuity, it is important to address this issue due to the visual distortion it can cause, which may substantially reduce patients’ quality of life. Findings from this review underscore the importance of selecting appropriate tamponade agents, removing residual fluid, and implementing intraoperative stabilization strategies to minimize retinal displacement. SO tamponades may be particularly beneficial for patients at risk of displacement, and the intraoperative use of PFCL to enhance retinal stability may help reduce visual distortions, especially in complex cases. Postoperative head positioning protocols remain critical for patient management, as adherence to specific positioning recommendations can minimize displacement risks and optimize visual outcomes. This review is subject to limitations common in systematic reviews of surgical interventions. Variability in study methodologies, sample sizes, and outcome measures among the included studies limits the generalizability of the findings. Casswell et al. noted that in their series they had images that had visible outer retinal folds, which presumably accompanied retinal shift, but no obvious characteristic findings on FAF images. Subsequently, they presumed that not all cases of retinal vessel shift can be obtained by imaging modalities [17]. Only one study provided long-term follow-up data on retinal displacement and its impact on quality of life, restricting our understanding of how retinal positioning affects functional vision over time. The variation in sample sizes, ranging from 20 to 239 patients in each study, can significantly impact the comparability of the overall conclusions. Studies with smaller sample sizes may lack sufficient statistical power, leading to less reliable conclusions. Furthermore, small sample sizes are more prone to extreme values, which can disproportionately affect study results and conclusions. In the presented studies, only four have a sample size greater than or equal to 70, which is another limitation, and demonstrates the need for further research. Additionally, the lack of representation of specific patient populations further limits the applicability of these results. Addressing these issues through targeted research will be essential to refine surgical protocols to meet the diverse needs of patients. Future research should focus on optimizing intraoperative and postoperative protocols to reduce retinal displacement risks and address the gaps in the existing literature. Recent advancements in stimuli-responsive polymers have shown significant potential in addressing the challenges of retinal displacement post surgery. Nguyen and Lai (2020) emphasize the development of polymer-based systems capable of responding to physiological triggers, such as potential of hydrogen or temperature changes, to achieve controlled drug release and biomechanical support in ocular treatments. These smart materials, designed to enhance biocompatibility and functional integration, represent a promising innovation in tamponade agents. By providing dynamic adaptability within the vitreous cavity, these polymers could not only improve retinal stabilization but also reduce complications associated with traditional tamponade materials, such as inflammation and displacement. Future investigations into these responsive biomaterials may pave the way for multi-functional solutions that simultaneously address reattachment and therapeutic delivery needs, further optimizing postoperative outcomes in retinal detachment repair. The introduction of new tamponade agents, such as semifluorinated alkanes and advanced hydrogels, represents a promising development in vitreoretinal surgery. These agents aim to combine effective retinal stabilization with reduced adverse effects compared to conventional materials. Semifluorinated alkanes, for example, offer enhanced biocompatibility and lower risks of emulsification, while hydrogels mimic the viscoelastic properties of natural vitreous, potentially reducing displacement risks and postoperative complications. As these materials advance through clinical trials, they hold the potential to revolutionize the standard of care in retinal detachment repair by addressing the limitations of current tamponade options [24,25]. Longitudinal studies examining the long-term impact of retinal displacement on vision and quality of life would provide valuable insights. Future studies should also consider under-represented populations, such as highly myopic patients, to develop targeted protocols that address the unique challenges associated with retinal reattachment in these groups. This review provides a comprehensive synthesis of studies on retinal displacement following PPV for RRD, yet certain limitations should be noted. First, the review was not registered in a systematic review database, which may limit the reproducibility and transparency of the initial review protocol. Additionally, only studies published in English were included, potentially introducing language bias and excluding relevant studies in other languages. Another limitation arises from the reliance on published data alone, which could introduce publication bias, as studies with null or negative findings may be less likely to be published.

## 5. Conclusions

RD has become a prominent subject of recent research. This systematic review consolidates current evidence on RD following PPV for RRD. Given that this phenomenon is relatively recent, there are few comprehensive reports in the literature, while numerous studies that have investigated risk factors for RD findings have been inconsistent. This review underscores the importance of postoperative and intraoperative management. Despite our focus on high-quality studies, limitations remain such as variability in methodologies and under-represented patient populations. Long-term studies with larger cohorts are needed to strengthen the evidence base and refine clinical guidelines. Future research should focus on optimizing intraoperative and postoperative protocols to minimize retinal displacement and improve functional outcomes.

## Figures and Tables

**Table 1:** ROBINS-I for non-randomized studies of interventions.

	D1	D2	D3	D4	D5	D6	D7	Overall
Shiragami et al., 2010 [14]	Moderate	Low	Moderate	Serious	Low	Moderate	Moderate	Serious
Chelazzi et al., 2021 [4]	Serious	Moderate	Low	Serious	Moderate	Serious	Moderate	Serious
Dell’Omo et al., 2017 [8]	Moderate	Low	Low	Moderate	Low	Moderate	Low	Moderate
Fillippelli et al., 2021 [15]	Serious	Moderate	Low	Moderate	Moderate	Serious	Moderate	Serious
Casswell et al., 2019 [16]	Moderate	Low	Low	Serious	Moderate	Moderate	Low	Serious
Dell’Omo et al., 2012 [5]	Moderate	Low	Low	Moderate	Low	Moderate	Low	Moderate
Caswell et al., 2020 [17]	Moderate	Low	Low	Serious	Moderate	Moderate	Low	Serious
Codenotti et al., 2013 [7]	Moderate	Moderate	Low	Serious	Moderate	Serious	Moderate	Serious
Dell’Omo et al., 2013 [3]	Moderate	Low	Low	Moderate	Low	Moderate	Low	Moderate

Domains: D1: Bias due to confounding. D2: Bias due to selection. D3: Bias due to classification of interventions. D4: Bias due to deviations from intended interventions. D5: Bias due to missing data. D6: Bias in measurement of outcomes. D7: Bias in selection of reported result.

**Table 2:** Cochrane Risk of Bias.

	D1	D2	D3	D4	D5	Overall
Schawkwat et al., 2021 [18]	High risk	Some concerns	High risk	Low risk	Some concerns	High risk
Guber et al., 2019 [19]	High risk	Low risk	Low risk	Low risk	Low risk	Moderate risk

Domains: D1: Bias arising from the randomization process. D2: Bias due to deviations from intended interventions. D3: Bias due to missing outcome data. D4: Bias in measurement of the outcome. D5: Bias in selection of the reported result.

**Table 3:** Summary of systemic review findings for primary outcomes, associated factors, and secondary study outcomes with GRADE quality of evidence evaluation.

Outcome	No. of Studies	Result	Quality (GRADE)	Comments
Retinal Displacement	11	Reported rates of retinal displacement vary significantly, ranging from 4.5% to 62.8%. Gas tamponade generally had higher displacement rates than silicone oil.	Low	Although widely reported, moderate-to-serious bias in study designs and inconsistency in reported rates reduce confidence in these findings.
Direction of RD	4	Downward displacement is the most common pattern (Dell’Omo et al., 2017 [8]: 88.6%). Some upward displacement reported, but no consistent correlation with detachment location.	Moderate	Findings are consistent across studies, but limited sample sizes prevent higher confidence.
Impact of Tamponade Agents	4	Silicone oil had significantly lower displacement rates (Filippelli et al.: 4.5% [15]; Dell’Omo et al., 2017: 14.3% [8]) compared to gas tamponade.	Moderate	Consistent across studies, with clear statistical support.
Preoperative Macula Status	7	Macula-off status associated with higher risk of displacement. Patients with macula-on detachments had minimal displacement.	Low	Moderate bias in most studies assessing macula-off impacts, though findings are consistent.
BCVA	4	Retinal displacement did not consistently correlate with postoperative BCVA.	Low	Imprecision due to underpowered studies limits reliability of findings.
Visual Distortion	4	Associated with retinal displacement.	Moderate	Consistently reported but lacks uniform definitions and measurement tools across studies.
Extent of Detachment	7	Quadrants involved in detachment did not consistently impact displacement (significant in only 1 study: *p* = 0.019).	Low	Data inconsistency and lack of focus on this factor limit confidence.
PVR	4	PVR grading inconsistently reported; one study (Filippelli et al. [15]) noted retinal displacement in cases with PVR B but lacked statistical power.	Very low	Insufficient data to draw robust conclusions.
Postoperative Positioning	4	Face-down positioning reduces displacement in gas tamponade cases (Chelazzi et al. [4], Casswell et al. [16])	Moderate	Findings are consistent but based on limited studies.
PFCL	4	PFCL use associated with smaller macular shifts in two studies.	Low	Limited and inconsistent evidence on PFCL’s effects.

**Table 4:** Summary of included study characteristics.

Study Type	Author, Year	Number of Centers	Follow-Up Duration	Age in Years (Mean SD)	PVR Grade	Number of Pseudophakic (%)	Number of Men (%)	Presence of Retinal Displacement (%)	Total Number of Patients	Macular Status	Use of Tamponade Agent	Postoperative Posture	Number of Quadrants of RRD Affected	Use of PFCL	Imaging Modality
Prospective randomized study	Casswell et al., 2020 [17]	Multiple	8 weeks and 6 months	60.8	None-230 PVR B 7 PVR C 2	N/R	71.5	42–58%	239	Off	SF6-110 C2F6-69 C3F8-32	Face down or support the break for 24 h followed by support-the-break positioning for 6 days	In hours 6–5.5	3	FC
Prospective randomized controlled trial	Schawkat et al., 2019 [18]	Single	3 weeks with SF6 6 weeks with C3F8	69	excluded	N/R	72	34%	50	Off	C3F8-88% SF6-12%	Log-roll with face-down position or lying flat on back for 6 h, then support-the-break end position	1—2 2—31 3—17 4—8	25	cSLO
Prospective observational study	dell’Omo et al., 2017 [8]	Multiple	12 months	60.9 ± 11.7	A-B	63.2	68	35.2%	125	Off	SF6-77.6% SO-22.4%	Prone 24 h after surgery	2—53.6% 3—20.8% 4—25.6%	80%	cSLO
Prospective observational study	dell’Omo et al. 2013 [3]	Single	4 weeks	64.3 ±3.5	N/R	80	60	35%	20	Off	SF6 20%	Face-down positioning for 2 h immediately after surgery	Mean 2.95 ± 0.75	No	cSLO
Retrospective Study	Chelazzi et al., 2021 [4]	Multiple	3 weeks SF6 6 weeks C3F8 1 month SO	59.1 ± 10.3	A-B	77.3	59	28.2%	75	On: 45.3% Off: 54.7%	20%SF6-20%/12%C3F8-58.7% Silicone Oil 8% Air 13.3%	Supine in macula-off In case of SRF, prone for few hours and then supine or support the break	Superior 45.3% inferior 18.7% superior + inferior 36%	No	cSLO
Retrospective observational Study	Filippelli et al., 2021 [15]	Single	3 months	65.8 + 11.4	A: 15.9% B: 63.6% C: 20.5%	34	66	4.5%	44	Off	Silicone Oil	Face-down position for 24 h	3: 38.6% 4: 61.4%	68.2%	cSLO
Prospective interventional case series	Shiragami et al., 2010 [14]	Single	6 months	60	Excluded	N/R	60.5	62.8%	43	On: 44% Off: 56%	20% SF6	Patients sat for a few minutes postoperatively before assuming a prone position for 7 to 10 days	1—2 2—31 2—8 4—2	30	FC
Prospective randomized controlled study	Guber et al., 2019 [19]	Single	6 months	69	Excluded	32%	72	34.00%	49	Off	SF6-88, C3F8-12	Log-roll 52% or Flat position: 24%	1—2, 2—23 3—17 4—8	25	cSLO
Prospective study	dell’Omo et al., 2012 [5]	Single	1 month	59.9 ± 8.5	N/R	27	82	10%	33	On: 27 Off: 73	SF6	N/R	4—3 3—9 2—18 1—3	45%	cSLO
Prospective study	Casswell 2019 [16]	Single	8 weeks	60.3 ± 7.7	N/R	30	75.7	61.4% (43/70) in FC 52.8% (37/70) cSLO	70	Off	Gas	Face-down 45% or bubble to break 55% (randomized) for 24 h	N/R	No	FC and cSLO
Prospective study	Codenotti 2013 [7]	Single	3 moths	57.1 ± 9.4	N/R	39%	65.2	52.2%	23	Off: 10 On: 13	C3F8 6% 14 eyes SO-9 eyes	1 week	1—6 2—10 3—3 4—4	Yes	FC

## Abbreviations

RRD	rhegmatogenous retinal detachment
PFCL	perfluorocarbon liquid
SO	silicone oil
OCT	optical coherence tomography
PPV	pars plana vitrectomy
PVR	proliferative vitreoretinopathy
FAF	fundus autofluorescence imaging
BCVA	best corrected visual acuity
FC	fundus camera
cSLO	confocal scanning laser ophthalmoscopy
RPE	retinal pigment epithelium
SRF	subretinal fluid
N/R	not reported
C3F8	perfluoropropane
SF6	Sulfur Hexafluoride
